# Bioassay-Guided Identification of the Antiproliferative Compounds of *Cissus trifoliata* and the Transcriptomic Effect of Resveratrol in Prostate Cancer Pc3 Cells

**DOI:** 10.3390/molecules26082200

**Published:** 2021-04-11

**Authors:** Luis Fernando Méndez-López, Pierluigi Caboni, Eder Arredondo-Espinoza, Juan J. J. Carrizales-Castillo, Isaías Balderas-Rentería, María del Rayo Camacho-Corona

**Affiliations:** 1 Laboratorio de Química Farmacéutica, Facultad de Ciencias Químicas, Universidad Autónoma de Nuevo León, Ciudad Universitaria, San Nicolás de los Garza, Nuevo León 66451, Mexico; eder.arredondosp@uanl.edu.mx (E.A.-E.); juan.carrizalescstl@uanl.edu.mx (J.J.J.C.-C.); isaias.balderasrn@uanl.edu.mx (I.B.-R.); 2 Centro de Investigación en Nutrición y Salud Publica, Facultad de Salud Pública y Nutrición, Universidad Autónoma de Nuevo León, Monterrey, Nuevo León 66460, Mexico; 3 Dipartamento Scienze della vita e dell’ambiente, Università degli Studi di Cagliari, Cittadella Universitaria, Monserrato, 09042 Cagliari, Italy; caboni@unica.it

**Keywords:** *Cissus trifoliata*, bioactive compounds, antitumor, PC3, resveratrol, microarrays

## Abstract

The bioassay-guided fractionation of a CHCl_3_-MeOH extract from the stems of *Cissus trifoliata* identified an active fraction against PC3 prostate cancer cells. The treatment for 24 h showed an 80% reduction in cell viability (*p* ≤ 0.05) by a WST-1 assay at a concentration of 100 μg/mL. The HPLC-QTOF-MS analysis of the fraction showed the presence of coumaric and isoferulic acids, apigenin, kaempferol, chrysoeriol, naringenin, ursolic and betulinic acids, hexadecadienoic and octadecadienoic fatty acids, and the stilbene resveratrol. The exposure of PC3 cells to resveratrol (IC_25_ = 23 μg/mL) for 24 h induced significant changes in 847 genes (Z-score ≥ ±2). The functional classification tool of the DAVID v6.8 platform indicates that the underlying molecular mechanisms against the proliferation of PC3 cells were associated (*p* ≤ 0.05) with the process of differentiation and metabolism. These findings provide experimental evidence suggesting the potential of *C. trifoliata* as a promising natural source of anticancer compounds.

## 1. Introduction

Despite recent advances in therapy used for the clinical management of metastatic prostate cancer, survival remains lower than 30% and the disease is the second leading cause of cancer mortality in men [1]. The use of natural sources of phytochemicals is an emerging non-toxic therapeutic strategy to prevent, delay, or treat prostate cancer [2,3]. The genus *Cissus* belongs to the Vitaceae family of plants and several studies have shown that extracts from their stems can inhibit the proliferation of different types of cancer cells in vitro [4]. In our preliminary screening, hexane, chloroform, and aqueous extracts from the stems of *C. trifoliata* showed cytotoxic activity in vitro against cancer cells from the liver, breast, cervix, lung, and prostate. The metabolic profile of the stems of *C. trifoliata* was characterized by a high content of terpenes, sterols, flavonoids, and stilbenes with anticancer activities [5]. Additionally, our results were consistent with the traditional use of *C. trifoliata* in the Mexican and South American ethnomedical systems for the management of tumors [6,7,8]. In this contribution following a bioassay-guided approach, a bioactive fraction against PC3 cells was identified through the column chromatography of a CHCl_3_-MeOH extract from the stems of *C. trifoliata*. Thirteen phytochemicals were identified by HPLC-QTOF-MS in the fraction and resveratrol was selected for the evaluation of its mechanism of action against PC3 cells. The microarrays showed that resveratrol treatment induces the regulation of genes tightly associated with differentiation and metabolism. Our results provide insight on the antitumor activity of *C. trifoliata*, of which the bioactive content might have potential therapeutic value in prostate cancer management, and the transcriptomic profile induced by resveratrol treatment in PC3 cells is reported for the first time.

## 2. Results

### 2.1. Cytotoxicity of the Fractions from the CHCl_3_-MeOH Extract

**Figure 1:**
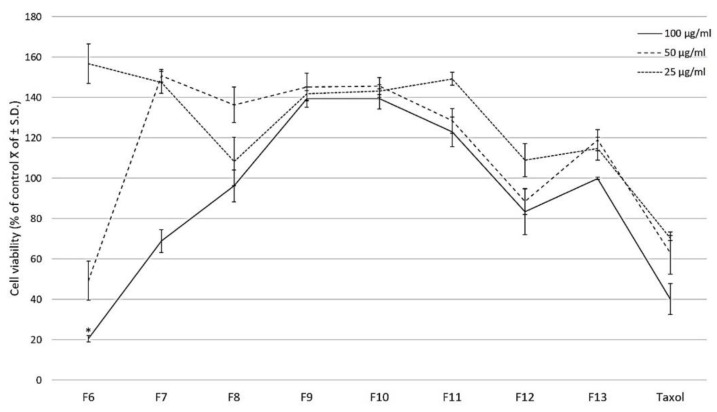
Effect of fractions of the CHCl_3_-MeOH extract from the stems of *C. trifoliata* and Taxol on PC3 cell proliferation. At 24 h after seeding, three concentrations of the fractions were added (25, 50, 100 μg/mL) with untreated cells as the negative control. The effect on cell viability was measured with the WST-1 assay and the results are presented as the percentage of the mean of control viable cells ± standard deviation. Fraction 6 significantly reduced cell proliferation at 100 μg/mL. Error bars are standard deviations and a significant difference is indicated as * *p* ≤ 0.05, ANOVA-LSD test.

The column chromatography fractionation of the CHCl_3_-MeOH extract from the stems of *C. trifoliata* resulted in 210 fractions that were combined according to thin layer chromatography (TLC) behavior in thirteen fractions. The pooled fractions that eluted from 70% of ethyl acetate (F6 to F13) were evaluated against the prostate cancer cell line PC3 by a WST-1 assay. The treatment for 24 h with three concentrations (25, 50, and 100 μg/mL) showed that fraction F6 significantly reduced 80% of cell proliferation at 100 μg/mL (*p* ≤ 0.05 ANOVA-LSD test) (Figure 1).

### 2.2. Chemical Analysis of the Active Fraction F6

**Figure 2:**
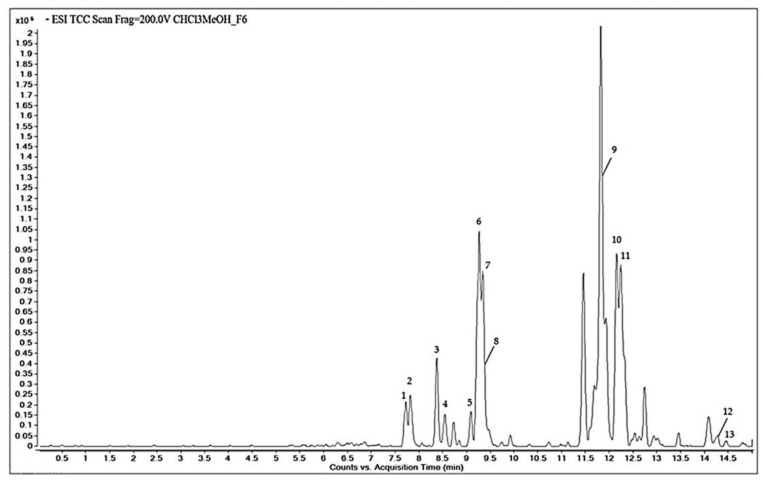
Total compound chromatogram obtained by HPLC-QTOF-MS of fraction F6 of CHCl_3_-MeOH stem extract of *C. trifoliata*.

The chemical constituents of the F6 fraction of the CHCl_3_-MeOH extract from the stems of *C. trifoliata* were analyzed by HPLC-QTOF-MS in negative scan mode. A representative chromatogram is shown (Figure 2).

Based on the accurate mass spectral data and the molecular formula, 13 bioactive compounds were tentatively identified in the METLIN database. Table 1 summarizes the molecules along with the retention time, the area %, the experimental m/z, and the molecular formula. The qualitative chemical profile of the active fraction includes the phenolic compounds trans-p-coumaric acid and isoferulic acid, the flavonoids dihydrokaempferol, apigenin, kaempferol, chrysoeriol, and naringenin, and the stilbene resveratrol. The cytotoxic fraction also contains the triterpenes hydroxyursolic acid, ursolic acid, and betulic acid, and hexadecadienoic and octadecadienoic fatty acids.

### 2.3. Growth Inhibition of PC3 Cells by Resveratrol

**Figure 3:**
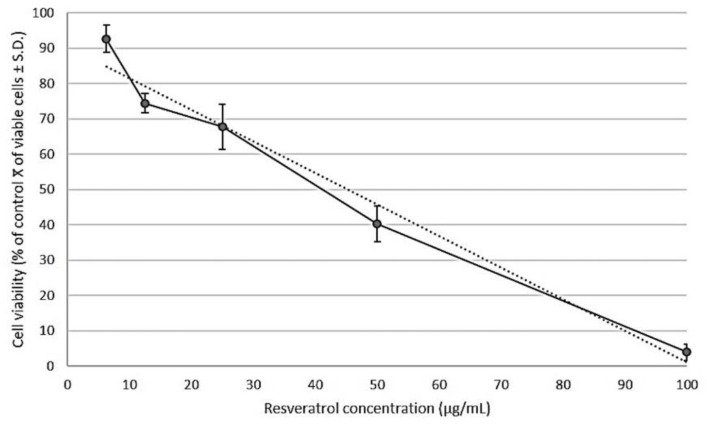
Cell viability was determined after 24 h of treatment with serial concentrations of resveratrol (6.25, 12.5, 25, 50, 100 μg/mL). The growth inhibition was measured with a WST-1 assay. The percentage of growth was calculated with 100% representing control cells treated with 0.6% DMSO alone. The results are presented as the average ± standard deviation from triplicate experiments and were subjected to linear regression (R^2^ = 0.97) for determination of the half inhibitory concentration (IC_50_). The calculated IC_50_ value of resveratrol in PC3 cells was 46 μg/mL.

The antiproliferative activity of resveratrol in the human prostate cancer cell line PC3 was examined by a WST-1 assay. Exponentially dividing cells were exposed to increasing concentrations of resveratrol of 6.25, 12.5, 25, 50, 100 μg/mL for 24 h. Resveratrol caused marked growth inhibition of PC3 cells, in a dose-dependent manner, with a calculated IC_50_ value of 46 µg/mL (Figure 3).

### 2.4. Transcriptional Effects of Resveratrol on PC3 Cells

The molecular mechanisms of non-lethal concentrations of resveratrol on the inhibition of the proliferation were explored by microarrays. The differentially expressed genes with a Z-score ≥ (±2) were analyzed to infer the genome-wide changes undergone by PC3 cells after exposure to the IC_25_ of resveratrol (23 μg/mL) for 24 h. The functional enrichment analysis of the list of the 847 genes was performed with the functional classification tool of the DAVID v6.8 platform. Resveratrol upregulated 526 genes in PC3 cells related to nuclear events of transcriptional regulation involving the homeobox proteins in the process of differentiation, effects in the mitochondrial membrane, and the activity of hydrolases and ubiquitin ligases. On the other hand, the 322 downregulated genes were associated with components of the membrane, the cytoplasm, and functions of signal transduction, receptors, and transport (Table 2 and Supplementary Materials).

## 3. Discussion

The fractionation of a CHCl_3_-MeOH extract from the stems of *C. trifoliata* led to the identification of a bioactive fraction that inhibits the proliferation of the PC3 cells in vitro. The content of the active fraction was tentatively identified in the METLIN database and showed the presence of compounds with proved anticancer qualities [9,10,11,12,13]. Among them, apigenin, ursolic acid, betulinic acid, and resveratrol showed relevant activities against prostate cancer in murine models and PC3 cells in vitro. Apigenin decreases cell viability and induces apoptosis of PC3 cells in vitro by increasing the levels of cytochrome C and suppressing the antiapoptotic proteins XIAP, survivin, Bcl-xL, and Bcl-2 [14]. Apigenin also produces downmodulation of HIF-1α and Akt pathways in PC3 cells which are essential for the progression and invasion of tumors [15]. Furthermore, the administration of apigenin inhibited tumor growth in mice with prostate cancer xenografts of PC3 cells [14]. The apoptotic effects of ursolic acid against PC3 cells are mediated by the downregulation of Bcl-2 [16], whereas its antitumor effects in prostate cancer growth in nude mice were associated with the suppression of NF-κB and STAT3 pathways [17]. In the case of betulinic acid, its inhibitory effects in the proliferation of PC3 cells in vitro were mediated by degradation of the cyclins A, B1, D1, and the cyclin-dependent kinases 1, 2, 4. Interestingly, the treatment with betulinic acid of the transgenic TRAP mice model of metastatic prostate cancer resulted in tumor growth inhibition and apoptosis mediated by caspase-3 activation and downregulation of the androgen receptor [18]. The effects of resveratrol on apoptosis and invasiveness of PC3 cells were through miR-21 and Akt negative modulation [19]. Further, in a recent report, resveratrol reduced the proliferation of PC3 cells by interfering with their metabolism and respiration through negative modulation of HIF-1α [20]. Resveratrol treatment also inhibited tumor growth in vivo in a model of immunodeficient male mice subcutaneously injected with PC3 cells [19]. Hence, the compounds in the bioactive fraction might act in synergy and contribute to the antitumor properties associated with *C. trifoliata* in traditional medicine. According to other studies, the content of terpenes, flavonoids, and stilbenes plays a major role in the anticancer properties of plants from the genus *Cissus* [5,21,22]. For example, from the fractionation of the ethanolic extract of the aerial part of *C. quadrangularis*, a bioactive fraction against MCF7 breast cancer cells in vitro with a high content of the flavonoids quercetin and rutin was obtained [21]. The hydroalcoholic leaf extract from *C. sicyoides* showed inhibition of Ehrlich carcinoma in mice and the bioactivity was related to the content of sitosterol and resveratrol [22]. Some of the constituents of the bioactive fraction of *C. trifoliata* have been isolated from the stems of other species of the genus *Cissus*, such as ursolic and betulic acids in *C. assamica* [23] and apigenin in *C. digitata* [24], whereas kaempferol [25] and resveratrol have been found in *C. quadrangularis* [26]. The plants from the genus *Cissus* belong to the family Vitaceae [27], hence it is expected that the bioassay-guided study of the stems of *Vitis vinifera* against MCF7 cells would have similar results. The fractionation of the ethyl acetate extract identified oleanolic acid, betulinic acid, and resveratrol as the most antiproliferative constituents in the stems of the plant [28]. Given the narrow distribution of resveratrol in the plant kingdom and the lack of reports of its transcriptomic effects in PC3 cells, the stilbene was selected for the microarray study. The gene expression changes undergone by the PC3 cells exposed for 24 h to resveratrol IC_25_ were measured with a microarray that contains the entire human genome sequence. The exposure to sublethal doses of resveratrol anticipates the identification of non-cytocidal mechanisms of action since this type of approach has been useful in the elucidation of novel antitumoral activities of other well-known anticancer compounds, such as the anti-metastatic properties of apigenin [29]. The functional classification of the 526 upregulated genes in PC3 cells by the treatment with resveratrol indicates major effects in nuclear events of transcriptional regulation that affect the process of differentiation. Resveratrol upregulated the expression of 44 transcription factors that orchestrate cell fate decisions. Many of them belong to the homeobox genes that are deregulated in cancer but that also are expressed during normal development [30]. Resveratrol induced the expression of H6 family homeobox 2 (HMX2), homeobox A3 (Hox-A3), homeobox D12 (Hox D12), msh homeobox 2 (MSX-2), POU class 4 homeobox 2 (POU4F2), PBX/knotted homeobox 1 (PKNOX1), and lim homeobox 8 (LHX8). Although the role of homeobox proteins in prostate cancer remains largely unknown, the transcription factors containing the domain POU are expressed in normal prostatic epithelial cells and are related to hormone dependency for growth and proliferation [31]. The upregulation of POU4F2 suggests the reactivation of differentiation in response to resveratrol since PC3 cells normally exhibit androgen independence and a lack of an epithelial phenotype [32]. Consistent with the previous assumption, the homeobox transcription factor Nanog was among the 322 genes downregulated by resveratrol treatment. Its expression is endogenously detected in high levels in PC3 cells and confers to them an undifferentiated phenotype with increased resistance to conventional antineoplastic agents and intrinsic tumor-initiation phenotype and metastatic capabilities [33]. Importantly, the downregulation of Nanog abolishes the proliferation of PC3 cells in vitro by non-cytotoxic mechanisms and inhibits the formation of prostatic cancer in vivo by the promotion of a differentiated phenotype with increased formation of adheren junctions [34]. The tight junction proteins and cell adhesion molecules control cellular proliferation and are considered biomarkers of epithelial differentiation and barriers for tumorigenesis [35]. The genes that were upregulated by resveratrol treatment in the PC3 cells that belong to this class are the tight junction protein ZO-2 (TJP2), gap junction alpha-1 protein (GJA1), tight junction partitioning defective 6 homolog gamma Gap (PARD6G), gap junction alpha-10 protein (NP115991), unconventional myosin-VIIb (MYO7B), cell adhesion molecules syndecan-2 cell adhesion molecule (SDC2), high-affinity immunoglobulin gamma Fc receptor (FCGR1A), and necdin (NDN). Moreover, the effect of resveratrol enhancing the expression of cell adhesion molecules has been previously described as a mechanism for the prevention of liver carcinogenesis induced by chemicals [36]. Most of the 322 genes downregulated by resveratrol are associated with components and processes of the membranes and mitochondrial metabolism, including the mitochondrial cytochrome CYB561D2, NAD(P) transhydrogenase (NNT), NADPH P450 reductase (POR), c oxidase subunit 1 (MT-CO1), palmitoyltransferase specific to HRas (ZDHHC9), fatty acid amide hydrolase (FAAH2), very-long-chain 3-hydroxyacyl-CoA dehydratase (PTPLA), long-chain fatty acid transport protein 1 ligase (SLC27A1), acyl-coenzyme A thioesterase 11 (ACOT11), tricarboxylate mitochondrial transport protein (SLC25A1), sarcosine mitochondrial dehydrogenase (SARDH), and creatine kinase M-type (CKM). Altogether, these changes in expression indicate the disruption of cancer cell metabolism by resveratrol, particularly affecting mitochondrial functions [37]. In a recent report, resveratrol was shown to slow PC3 cell growth in vitro by interfering with glucose fermentation and promoting respiration [20]. Moreover, resveratrol exposure was previously linked with morphological changes in the mitochondria and epithelial differentiation in a colon carcinoma cell line (HCT116) [38]. Even though those changes were detected by microscopy, they are consistent with our major findings in the microarray study of PC3 cells exposed to resveratrol. From our results and the evidence referenced above, it seems plausible that resveratrol might play a major role in the antitumor effects of *C. trifoliata* by the induction of cell differentiation in cancer cells. Furthermore, three components of the fraction F6 display similar anticancer activities. For example, ursolic acid leads to the maturation of teratocarcinoma stem cells [39], kaempferol causes the differentiation of colon cancer cells and restores their morphology [40], and apigenin induces the full commitment of chronic myeloid leukemia cells into erythrocytes [41]. These data provide evidence for the potential therapeutic effect of *C. trifoliata* in the carcinogenesis of the prostate and are useful for further studies to increase the understanding of the molecular mechanisms underlying the ethnomedical use of this plant against tumors.

## 4. Materials and Methods

### 4.1. Plant Material and Extraction

*C. trifoliata* was collected and identified by trained biologist Mauricio González Ferrara in Rayones, Nuevo León, Mexico (latitude, 25.0167°, longitude: −100.05°, altitude: 900 m) on 10 October 2018. A voucher (027499) specimen was deposited in the Department of Botany of Universidad Autonóma de Nuevo León. Dried and ground stems (756 g) were subjected to extraction by maceration. The solvents used for maceration and column chromatography were chloroform (CHCl_3_) purity 98.8%, methanol (MeOH) purity 99.9%, ethyl acetate 99.9%, and hexane purity 98.99% (Baker, Phillipsburg, NJ, USA). The solvent mixture of CHCl_3_-MeOH (1:1, *v/v*) for maceration was added 4 times, for 24 h each (9 L). The extract was filtered and concentrated using a rotary evaporator at 40 °C (V300, Buchi, Flawil, Switzerland). Twenty-four grams of dry extract were kept at 4 °C until use.

### 4.2. Column Chromatography

The fractionation of the 24 g of CHCl_3_-MeOH extract from the stems of *C. trifoliata* was carried out with column chromatography using silica gel of 63–200 μm (Sigma-Aldrich, St. Louis, MO, USA) as the stationary phase in a 1:20 relation (480 g) and hexane, ethyl acetate, and methanol in the gradient as the mobile phase. A total of 210 fractions (20 mL each) were collected and pooled based on their TLCprofile into thirteen fractions. The fractions were airdried and weighed on an analytical balance (entris224, Sartorius, Goettingen, Germany) (Table 3). Fractionation was monitored with TLC of silica gel 60 F254 (Merck Fluka, Darmstadt, Germany) using visible and ultraviolet light at 254 nm and 365 nm with a Spectroline EF160C lamp (Spectronics, Westbury, NY, USA) and stained with Ce(SO_4_)_2_/H_2_SO_4_ solution.

### 4.3. Cell Culture and Cytotoxic Assay

The prostate adenocarcinoma cell line (PC3, CRL-1435) was purchased from the American Type Culture Collection (ATCC). Cells were grown in RPMI 1640 medium (Invitrogen, Thermo Fisher Scientific, Inc., Waltham, MA, USA), supplemented with 10% fetal bovine serum (FBS), 2 mM L-glutamine, 40 µg/mL gentamicin, and penicillin–streptomycin (100 U/mL penicillin and 100 µg/mL streptomycin). The culture was incubated at 37 °C in a humidified atmosphere of 5% CO_2_. The fractions F1–F5 were composed mainly of alkanes, fatty acids, esters, and sterols, hence their evaluation was neglected since we previously reported the cytotoxicity of the lipidic content of *C. trifoliata* stems against PC3 cells [5]. The fractions F6-13 of the CHCl_3_-MeOH extract and the positive control Taxol were dissolved in DMSO (≤0.6%) in supplemented culture medium. To determine the cytotoxic effect of the fractions, 5000 cells per well were seeded in a 96-well cell culture plate and treated with three concentrations of the fractions and Taxol (25, 50, 100 μg/mL). The PC3 cells were exposed for 24 h at 37 °C with 5% CO_2_ and the number of viable cells was determined using WST-1 reagent (Sigma-Aldrich, St. Louis, MO, USA), following the manufacturer’s instruction. Cell viability was determined by absorbance at 450 nm using an automated ELISA reader (Benchmark Scientific, Sayreville, NJ, USA). Experiments were conducted in triplicate in three independent tests. Results were expressed as a percentage of the mean of control viable cells ± standard deviation. Statistical analysis was performed by an ANOVA-LSD test in IBM SPSS Statistics Version 20 and *p*-value ≤ 0.05 was considered significant.

### 4.4. HPLC-QTOF-MS Analysis

The active fraction F6 was analyzed by reverse-phase HPLC on an Agilent 1200 series LC system fitted with microchip technology using an Agilent Zorbax 300 SB-C18 5 µm, 43 mm × 75 µm (Agilent, Santa Clara, CA, USA). Acetonitrile, methanol, and formic acid were HPLC grade (Baker, Milan, Italy). Water was distilled and filtered through Milli-Q apparatus (Millipore, Milan, Italy) before use. A sample of 0.5 mg of fraction F6 was diluted in 250 mL methanol. The solution was filtered with Acrodisc syringe filters with a 0.45 µm PTFE membrane (Sigma-Aldrich, St. Louis, MO, USA). The HPLC conditions were as follows: flow rate, 0.4 μL/min; solvent A, 0.1% formic acid in water; solvent B, acetonitrile, and the gradient was from 5% to 100% B over 15 min. One microliter of the sample was then analyzed by electrospray ionization (ESI) in negative mode using an Agilent 6520 time of flight (TOF) MS. Mass spectrometric data were acquired in the range m/z 100–1000 with an acquisition rate of 1.35 spectra/s, averaging 10,000 transients. The source parameters were adjusted as follows: drying gas temperature 250 °C, flow rate 5 L/min, nebulizer pressure 45 psi, and the fragmentor voltage 150 V. Data acquisition and processing were done using Agilent MassHunter Workstation Acquisition software v. B.02.00. Based on the original acquisition files, a pre-processing step with MetAlign software was performed for automated baseline correction and alignment of all extracted mass peaks across all samples. ESI/QTOF MS data were then analyzed using the molecular feature extraction algorithm of the MassHunter Workstation software version B 03.01 Qualitative Analysis (Agilent Technologies, Santa Clara, CA, USA). Reliable interpretation of the accurate mass spectral data generated was carried out in the METLIN database for compound identification.

### 4.5. Determination of the IC_50_ of Resveratrol

The pure compound was purchased from Sigma-Aldrich (R5010). The IC_50_ of resveratrol was calculated in the PC3 cells by the WST-1 protocol previously described for the fractions. Cells were exposed to 6.25, 12.5, 25, 50, and 100 (µg/mL) of resveratrol for 24 h. Stocks were prepared with DMSO, and assays were conducted in triplicate in three independent tests. The IC_50_ values were calculated by regression analysis using Microsoft Excel software.

### 4.6. Microarrays

PC3 cells were treated in a 6-well plate with 10^6^ cells per well for 24 h with resveratrol at its IC_25_ (23 µg/mL) and 0.6% DMSO or as a negative control only with DMSO at the same concentration. After incubation, the total RNA was extracted from both cultures treated either with the compound or with the vehicle, using Trizol Reagent (Invitrogen, Carlsbad, CA, USA) following the manufacturer’s instructions. The RNA quality was assessed by agarose gel (1%) and total RNA was quantified in a NanoDrop 2000 spectrophotometer (Thermo Fisher Scientific, Waltham, MA, USA). RNA quantity was adjusted to 20 µg for both treated and untreated controls. The modified complementary DNA (cDNA possessing the nucleotide aminoallyl-uridine) was synthesized using the amino-allyl cDNA labeling kit (Invitrogen, Life Technologies, Grand Island, NY, USA). The fluorescent dyes Cy3 and Cy5 from CyDye Post-Labeling Reactive Dye Packs (GE Healthcare Life Sciences, Aylesbury, Buckinghamshire, UK) were used for labeling the cDNA. Each vial of fluorescent dye was dissolved in 3 µL of DMSO and mixed with one specific cDNA. A NucAway column AM10070 (Invitrogen, Carlsbad, CA, USA) was used to eliminate excess unbound fluorophore. The microarray hybridization was performed following the procedure described in the protocol by MYcroarray (Ann Arbor, MI, USA) on a human 35K chip, surveying a total of 35,764 genes (whole genome). The labeled cDNA of both treated and control samples was deposited on the chip and the latter was placed in a G2534A hybridization chamber (Agilent, Santa Clara, CA, USA). Then, following the hybridization step at 42 °C for 24 h, the chip was washed three times with SSPE 1x and once with SSPE 0.25x and finally dried by centrifugation. The chip was read by measuring absorbance at 555 nm for Cy3 and 647 nm for Cy5 using a Gene Pix 4000B scanner (Agilent Technologies, Santa Clara, CA, USA). The statistical analysis of data was performed using the GenArise package to obtain the Z-score value, negative or positive according to down- or upregulated gene expression after treatment with the compound. The lists of genes with a Z-score ≥ (±2) were analyzed for the functional annotation using the bioinformatic tool Database for Annotation, Visualization, and Integrated Discovery (DAVID) v6.8. The results provided by the DAVID algorithm classified genes according to their functions, and a *p*-value ≤ 0.05 was considered significant.

## 5. Conclusions

The bioassay-guided fractionation of the stems of *C. trifoliata* in the model for the study of prostate cancer in vitro in PC3 cells enabled the identification of an active fraction composed of pentacyclic triterpenes, phenolic compounds, unsaturated fatty acids, and the stilbene resveratrol. The analysis of microarrays indicates that resveratrol affects the proliferation of PC3 cells by inducing changes in their metabolism and differentiation. Our study suggests that *C. trifoliata* possess bioactive content that might have a potential therapeutic value in the management of prostate cancer.

## Figures and Tables

**Table 1:** HPLC-QTOF-MS analysis of fraction F6 of the CHCl_3_-MeOH extract from the stems of *C. trifoliata.*

Peak	RT (min)	Area (%)	Experimental m/z [M−H] ^−^	Molecular Formula	Tentative Compound Identification in METLIN Database
1	7.685	2.55	163.0401	C_9_H_8_O_3_	Trans-p-coumaric acid
2	7.908	3.03	193.0504	C_10_H_10_O_4_	Isoferulic acid
3	8.541	10.26	287.0569	C_15_H_12_O_6_	Dihydrokaempferol
4	8.567	3.44	227.0716	C_14_H_12_O_3_	Resveratrol
5	9.103	8.62	269.0434	C_15_H_10_O_5_	Apigenin
6	9.243	4.60	285.0404	C_15_H_10_O_6_	Kaempferol
7	9.345	10.74	299.0561	C_16_H_12_O_6_	Chrysoeriol
8	9.479	3.46	271.0612	C_15_H_12_O_5_	Naringenin
9	11.699	22.51	471.3475	C_30_H_48_O_4_	2-alpha hydroxyursolic acid
10	12.394	16.11	455.3515	C_30_H_48_O_3_	Ursolic acid
11	12.471	10.12	455.3422	C_30_H_48_O_3_	Betulinic acid
12	14.401	3.38	253.2161	C_16_H_28_O_2_	Hexadecadienoic acid
13	14.582	1.18	279.2348	C_16_H_32_O_2_	Octadecadienoic acid

**Table 2:** Functional annotation data provided by DAVID algorithm from the modulated genes by the treatment of resveratrol IC_25_ in PC3 cells. The genes were classified according to their functional annotation, *p*-value ≤ 0.05 was considered significant.

Functional Category	Effect of Resveratrol	%	*p*-Value
Transcription regulation	Upregulation	19.82	2.30 × 10^−3^
Nucleus	Upregulation	11.97	7.50 × 10^−5^
Membrane	Downregulation	9.14	1.70 × 10^−3^
DNA binding	Upregulation	6.82	3.10 × 10^−5^
Integral component of membrane	Downregulation	6.56	2.00 × 10^−2^
Plasma membrane	Downregulation	5.66	9.60 × 10^−3^
Homeobox	Upregulation	5.53	6.60 × 10^−4^
Cytoplasmic	Downregulation	5.02	3.70 × 10^−3^
Cell membrane	Downregulation	4.12	3.10 × 10^−2^
Hydrolase	Upregulation	3.86	3.80 × 10^−2^
Transport	Downregulation	3.47	1.20 × 10^−3^
ATP binding	Upregulation	3.22	5.90 × 10^−2^
Receptor	Downregulation	2.83	5.30 × 10^−3^
Transducer	Downregulation	1.93	4.30 × 10^−3^
Endoplasmic reticulum membrane	Downregulation	1.93	5.10 × 10^−3^
G-protein coupled receptor pathway	Downregulation	1.93	6.50 × 10^−3^
Cell differentiation	Upregulation	1.80	6.90 × 10^−2^
Homeodomain−like	Upregulation	1.54	4.50 × 10^−3^
Mitochondrial inner membrane	Upregulation	1.54	3.50 × 10^−2^
Ubiquitin protein ligase binding	Upregulation	1.29	1.80 × 10^−2^

**Table 3:** Column chromatography fractionation of the CHCl_3_-MeOH extract.

Fractions	Mobile Phase	Pooled Fractions	Weight (g)
1–28	Hexane 100%	F1	0.1499
29–36	Hexane/Ethyl acetate 85:15	F2	1.8797
37–45	Hexane/Ethyl acetate 80:20	F3	0.9695
46–54	Hexane/Ethyl acetate 70:30	F4	0.4827
55–72	Hexane/Ethyl acetate 60:40	F5	2.1402
73–108	Hexane/Ethyl acetate 30:70	F6	1.5938
109–117	Ethyl acetate 100%	F7	0.4561
118–128	Ethyl acetate/Methanol 80:20	F8	0.6795
129–144	Ethyl acetate/Methanol 70:30	F9	1.3392
145–168	Ethyl acetate/Methanol 50:50	F10	4.1135
169–186	Ethyl acetate/Methanol 30:70	F11	3.7527
187–198	Ethyl acetate/Methanol 20:80	F12	1.5929
199–210	Methanol 100%	F13	1.2244

## Data Availability

Not applicable.

## Supplementary Materials

The following are available online. Full results of microarrays, transcriptomic effect of resveratrol IC25, 24 h in PC3 cells Z-score ≥ (±2).

## Sample Availability

Samples of the compounds are not available from the authors.

## References

[1] Crawford E.D., Andriole G.L., Marberger M., Rittmaster R.S. (2010). Reduction in the risk of prostate cancer: Future directions after the Prostate Cancer Prevention Trial. Urology.

[2] Park E.J., Pezzuto J.M. (2002). Botanicals in cancer chemoprevention. Cancer Metastasis Rev..

[3] Li Y., Ahmad A., Kong D., Bao B. (2014). Recent progress on nutraceutical research in prostate cancer. Cancer Metastasis Rev..

[4] Bhujade A., Gupta G., Talmale S., Das S., Patil M. (2013). Induction of apoptosis in A431 skin cancer cells by *Cissus quadrangularis* Linn stem extract by altering Bax-Bcl-2 ratio, release of cytochrome c from mitochondria and PARP cleavage. Food Funct..

[5] Méndez-López L.F., Garza-González E., Ríos M.Y., Ramírez-Cisneros M., Alvarez L., González-Maya L., Sánchez-Carranza J.N., Camacho-Corona M.D.R. (2020). Metabolic Profile and Evaluation of Biological Activities of Extracts from the Stems of *Cissus trifoliata*. Int. J. Mol. Sci..

[6] Magana A., Gama Campillo L.M., Mariaca Méndez R. (2010). El uso de las plantas medicinales en las comunidades Maya-Chontales de Nacajuca, Tabasco, México. Polibotánica.

[7] Quiros-Moran D. (2009). Guide to Afro-Cuban Herbalism.

[8] Heinrich M. (2000). Ethnobotany and its role in drug development. Phytother. Res..

[9] Kong C.S., Jeong C.H., Choi J.S., Kim K.J., Jeong J.W. (2013). Antiangiogenic effects of p-coumaric acid in human endothelial cells. Phytother. Res..

[10] Qin Y., Cui W., Yang X., Tong B.J. (2016). Kaempferol inhibits the growth and metastasis of cholangiocarcinoma in vitro and in vivo. Acta Biochim. Biophys. Sin..

[11] Knowles L.M., Zigrossi D.A., Tauber R.A., Hightower C., Milner J.A. (2000). Flavonoids suppress androgen-independent human prostate tumor proliferation. Nutr. Cancer..

[12] Lim W., Park S., Bazer F.W., Song G.J. (2017). Naringenin-induced apoptotic cell death in prostate cancer cells is mediated via the PI3K/AKT and MAPK signaling pathways. J. Cell Biochem..

[13] Han K.-Y., Chen P.-N., Hong M.-C., Hseu Y.-C., Chen K.-M., Hsu L.-S., Chen W. (2018). Naringenin Attenuated Prostate Cancer Invasion via Reversal of Epithelial-to-Mesenchymal Transition and Inhibited uPA Activity. Anticancer Res..

[14] Shukla S., Fu P., Gupta S.J. (2014). Apigenin induces apoptosis by targeting inhibitor of apoptosis proteins and Ku70-Bax interaction in prostate cancer. Apoptosis.

[15] Mirzoeva S., Kim N.D., Chiu K., Franzen C.A., Bergan R.C., Pelling J.C. (2008). Inhibition of HIF-1 alpha and VEGF expression by the chemopreventive bioflavonoid apigenin is accompanied by Akt inhibition in human prostate carcinoma PC3-M cells. Mol. Carcinog..

[16] Kassi E., Papoutsi Z., Pratsinis H., Aligiannis N., Manoussakis M., Moutsatsou P. (2007). Ursolic acid, a naturally occurring triterpenoid, demonstrates anticancer activity on human prostate cancer cells. J. Cancer Res. Clin. Oncol..

[17] Shanmugam M.K., Rajendran P., Li F., Nema T., Vali S., Abbasi T., Kapoor S., Sharma A., Kumar A.P. (2011). Ursolic acid inhibits multiple cell survival pathways leading to suppression of growth of prostate cancer xenograft in nude mice. J. Mol. Med..

[18] Reiner T., Parrondo R., de las Pozas A., Palenzuela D., Perez-Stable C.J. (2013). Betulinic acid selectively increases protein degradation and enhances prostate cancer-specific apoptosis: Possible role for inhibition of deubiquitinase activity. PLoS ONE.

[19] Sheth S., Jajoo S., Kaur T., Mukherjea D., Sheehan K., Rybak L.P., Ramkumar V. (2012). Resveratrol reduces prostate cancer growth and metastasis by inhibiting the Akt/MicroRNA-21 pathway. PLoS ONE.

[20] Fonseca J., Moradi F., Maddalena L.A., Ferreira-Tollstadius B., Selim S., Stuart J.A. (2019). Resveratrol integrates metabolic and growth effects in PC3 prostate cancer cells-involvement of prolyl hydroxylase and hypoxia inducible factor-1. Oncol. Lett..

[21] Vijayalakshmi A., Kumar P., Sakthi Priyadarsini S., Meenaxshi C. (2013). In vitro antioxidant and anticancer activity of flavonoid fraction from the aerial parts of *Cissus quadrangularis* linn against human breast carcinoma cell lines. J. Chem..

[22] Lucena F.R., Almeida E.R., Aguiar J.S., Silva T.G., Souza V.M., Nascimento S.C. (2010). Cytotoxic, antitumor and leukocyte migration activities of resveratrol and sitosterol present in the hidroalcoholic extract of *Cissus sicyoides* L., Vitaceae, leaves. Rev. Bras. Farmacog..

[23] Chan Y.-Y., Wang C.-Y., Hwang T.-L., Juang S.-H., Hung H.-Y., Kuo P.-C., Chen P.-J., Wu T.-S. (2018). The constituents of the stems of *Cissus assamica* and their bioactivities. Molecules.

[24] Al-Said M.S., Khalifa A.S., Al-Azizi M.M. (1991). Flavonoids from Cissus digitata. Int. J. Pharm..

[25] Thakur A., Jain V., Hingorani L., Laddha K. (2009). Phytochemical Studies on *Cissus quadrangularis* Linn. J. Pharm. Res..

[26] Adesanya S.A., Nia R., Martin M.-T., Boukamcha N., Montagnac A., Païs M. (1999). Stilbene derivatives from *Cissus quadrangularis*. J. Nat. Prod..

[27] Rodrigues J.G., Lombardi J.A., Lovato M.B. (2014). Phylogeny of Cissus (Vitaceae) focusing on South American species. Taxon.

[28] Amico V., Barresi V., Chillemi R., Tringali C. (2008). Bioassay-Guided Isolation of Antiproliferative Compounds from Grape (*Vitis vinifera*) Stems. Nat. Prod. Commun..

[29] Souza R.P., Bonfim-Mendonça P.d.S., Gimenes F., Ratti B.A., Kaplum V., Bruschi M.L., Nakamura C.V., Silva S.O., Maria-Engler S.S., Consolaro M.E. (2017). Oxidative stress triggered by Apigenin induces apoptosis in a comprehensive panel of human cervical cancer-derived cell lines. Oxid. Med. Cell Longev..

[30] Abate-Shen C.J. (2002). Deregulated homeobox gene expression in cancer: Cause or consequence?. Nat. Rev. Cancer.

[31] Dennis J.H., Budhram-Mahadeo V., Latchman D.S. (2001). The Brn-3b POU family transcription factor regulates the cellular growth, proliferation, and anchorage dependence of MCF7 human breast cancer cells. Oncogene.

[32] Lang S.H., Hyde C., Reid I.N., Hitchcock I.S., Hart C.A., Gordon Bryden A., Villette J.M., Stower M.J., Maitland N.J. (2002). Enhanced expression of vimentin in motile prostate cell lines and in poorly differentiated and metastatic prostate carcinoma. Prostate.

[33] Jeter C.R., Liu B., Liu X., Chen X., Liu C., Calhoun-Davis T., Repass J., Zaehres H., Shen J., Tang D.G. (2011). NANOG promotes cancer stem cell characteristics and prostate cancer resistance to androgen deprivation. Oncogene.

[34] Jeter C.R., Badeaux M., Choy G., Chandra D., Patrawala L., Liu C., Calhoun-Davis T., Zaehres H., Daley G.Q., Tang D.G. (2009). Functional evidence that the self-renewal gene NANOG regulates human tumor development. Stem Cells.

[35] Runkle E.A., Mu D.J. (2013). Tight junction proteins: From barrier to tumorigenesis. Cancer Lett..

[36] Nielsen M., Ruch R.J., Vang O.J. (2000). Resveratrol reverses tumor-promoter-induced inhibition of gap-junctional intercellular communication. Biochem. Biophys. Res. Commun..

[37] Sareen D., Van Ginkel P.R., Takach J.C., Mohiuddin A., Darjatmoko S.R., Albert D.M., Polans A.S. (2006). Mitochondria as the primary target of resveratrol-induced apoptosis in human retinoblastoma cells. Investig. Ophthalmol. Vis. Sci..

[38] Mahyar-Roemer M., Katsen A., Mestres P., Roemer K. (2001). Resveratrol induces colon tumor cell apoptosis independently of p53 and precede by epithelial differentiation, mitochondrial proliferation and membrane potential collapse. Int. J. Cancer..

[39] Lee H.-Y., Chung H.-Y., Kim K.-H., Lee J.-J., Kim K.-W. (1994). Induction of differentiation in the cultured F9 teratocarcinoma stem cells by triterpene acids. J. Cancer Res. Clin. Oncol..

[40] Nakamura Y., Chang C.-C., Mori T., Sato K., Ohtsuki K., Upham B.L., Trosko J.E. (2005). Augmentation of differentiation and gap junction function by kaempferol in partially differentiated colon cancer cells. Carcinogenesis.

[41] Isoda H., Motojima H., Onaga S., Samet I., Villareal M.O., Han J.J. (2014). Analysis of the erythroid differentiation effect of flavonoid apigenin on K562 human chronic leukemia cells. Chem. Biol. Interact..

