# Supplementary material for: Bioassay-Guided Identification of the Antiproliferative Compounds of Cissus trifoliata and the Transcriptomic Effect of Resveratrol in Prostate Cancer Pc3 Cells
Source: Molecules. 2021 Apr 11;26(8):2200. doi: 10.3390/molecules26082200 (PMC8070146; doi:10.3390/molecules26082200)
Supplement: Supplementary file 1 [file molecules-26-02200-s001.pdf]

molecules

MDPI

Article

Bioassay-Guided Identification of the Antiproliferative Compounds of *Cissus Trifoliata* and the Transcriptomic Effect of Resveratrol in Prostate Cancer Pc3 Cells

Luis Fernando Méndez-López [1,2,*], Pierluigi Caboni [3], Eder Arredondo-Espinoza [1], Juan J.J. Carrizales-Castillo [1], Isaías Balderas-Rentería [1] and María del Rayo Camacho-Corona [1,*]

[1] Facultad de Ciencias Químicas, Universidad Autónoma de Nuevo León, Ciudad Universitaria, San Nicolás de los Garza, C.P. 66451, Nuevo León, México; eder.arredondosp@uanl.edu.mx; isaias.balderasrn@uanl.edu.mx; juan.carrizalescstl@uanl.edu.mx
[2] Centro de Investigación en Nutrición y Salud Publica, Facultad de Salud Pública y Nutrición, Universidad Autónoma de Nuevo León, Monterrey, C.P. 66460, Nuevo León, México; luis.mendezlop@uanl.edu.mx
[3] Dipartamento Scienze della vita e dell'ambiente, Università degli Studi di Cagliari, Cittadella Universitaria, Monserrato, C.P. 09042, Cagliari, Italy; caboni@unica.it
* Correspondence: luis.mendezlop@uanl.edu.mx (L.F.M.L.), maria.camachocn@uanl.edu.mx; (M.d.R.C.C); Tel.: +52-81-8329-4000-3414 (M.d.R.C.C)

Supplementary Information

Full gene list of microarrays Z-score ≥ (± 2). PC3 cells were treated for 24 hours with resveratrol at its IC_{25} (23 μg/mL) and 0.6% DMSO or as a negative control only with DMSO at the same concentration. The microarray hybridization was performed on a human 35K chip surveying a total of 35,764 gene (Mycroarray Company, Ann Arbor, MI, USA). Data was then submitted to statistical analysis performed using the GenArise package to obtain genes with Z-score ≥ (± 2) were selected to carry out the gene expression analysis.

Upregulated genes

N	Cy3	Cy5	Id	Symbol	Zscore
1	63	141	OPHSV0400013338	hsa-mir-218-1	2.455369572
2	64	139	OPHSV0400006408	ADAM29	2.155016066
3	65	139	H200000391	SDC2	2.032136009
4	65	141	H300000263	C18orf17	2.181449056
5	65	154	OPHSV0400010459	-	2.1545111
6	64	160	OPHSV0400011321	-	2.665415398
7	68	158	OPHSV0400009034	-	2.352875188
8	68	160	OPHSV0400001569	ZNF662	2.22004353
9	74	156	OPHSV0400006493	TMPRSS3	2.162812455
10	64	183	OPHSV0400012804	-	2.560573408
11	69	170	H200008409	SELK_HUMAN	2.007325284
12	66	181	OPHSV0400008275	-	2.265172144
13	79	156	OPHSV0400007963	-	2.174161672

14	74	167	OPHSV0400012439	-	2.97887194
15	70	179	H200003126	SCG3	2.797688874
16	76	165	H200004729	RP2	2.018798578
17	74	170	OPHSV0400002049	LOC727910	2.251182413
18	77	168	OPHSV0400008316	Q6ZP58_HUMAN	2.243854875
19	79	164	H2NC000009	-	2.013526345
20	63	215	AF159803_80	-	3.390259395
21	69	198	H200008150	ATP10D	2.428227379
22	72	191	OPHSV0400003763	-	2.507926038
23	72	191	OPHSV0400010759	-	2.517989181
24	78	179	OPHSV0400008365	-	2.620359394
25	79	180	H200006613	NMBR	2.037417243
26	73	197	H300016170	SPTBN5	2.636541355
27	78	185	OPHSV0400010568	-	2.379457592
28	85	172	OPHSV0400003247	CU081_HUMAN	2.390450155
29	71	208	OPHSV0400013192	-	3.389826437
30	82	181	H300007556	NFAM1	2.038146112
31	84	178	OPHSV0400005872	DFFA	2.433881211
32	89	170	OPHSV0400001691	-	2.146966748
33	88	172	OPHSV0400002825	KCNK18	2.263875821
34	85	180	H300009186	MAFA	2.490855921
35	90	173	H200013957	TLL2	2.063489645
36	90	173	OPHSV0400006496	ASPH	2.064705131
37	85	184	OPHSV0400006011	LRRC37A	2.197108914
38	86	182	H200021085	TMEM143	2.122885176
39	83	190	OPHSV0400006589	GLYATL2	2.448005069
40	82	194	OPHSV0400000821	FAM18B	3.155825612
41	91	177	H300008359	NDUFB3	2.185027808
42	84	193	OPHSV0400012555	-	2.617024552
43	91	180	OPHSV0400001887	-	2.15009463
44	81	205	M2NC000002	-	2.628856346
45	77	216	H300002242	TRIM7	3.043834255
46	81	207	H200012618	VPS4B	2.582367636
47	88	193	H300021108	C17orf78	2.132772725
48	89	192	H300001394	Q8N4T9_HUMAN	2.033234278
49	89	194	H300001600	LAGE3	2.313103974
50	88	197	OPHSV0400002103	-	2.46701652
51	83	211	OPHSV0400004609	Q9P1E4_HUMAN	3.041599584
52	81	218	H300016341	PIK3AP1	2.447619438
53	74	239	H200010978	VANGL1	3.016776533
54	86	208	OPHSV0400011291	-	2.318888395
55	90	199	OPHSV0400003813	-	2.106439149
56	87	206	H200004190	GRM1	2.411105277
57	95	192	AF247559_50	-	2.29726402
58	89	207	H300021115	Q7Z2F6_HUMAN	2.474436609
59	95	196	OPHSV0400010206	-	2.664280957
60	93	202	H300018580	RBM41	2.106879369
61	79	238	OPHSV0400003243	SRFBP1	3.012456613
62	93	204	H300018993	C14orf179	2.477658251
63	99	192	OPHSV0400003172	HMGB3	2.02528844
64	92	209	OPHSV0400009048	-	2.093463745
65	92	209	OPHSV0400009260	-	2.10099607
66	88	220	H200007186	FAM64A	2.255847198
67	94	207	H300005908	CCDC57	2.045693269

68	97	202	H200011810	ARRB1	2.265645886
69	95	207	OPHSV0400007307	-	2.241429842
70	96	206	H200014429	C20orf94	2.048243452
71	90	220	H200000071	POU4F2	2.729935785
72	66	303	H200010965	ZBTB32	3.369684838
73	82	246	OPHSV0400011123	-	2.69348632
74	93	217	OPHSV0400010106	-	2.086107916
75	101	202	OPHSV0400008628	-	2.083807491
76	98	210	OPHSV0400007144	NP_001017361.1	2.491130002
77	103	200	OPHSV0400012171	-	2.138368897
78	96	217	H300001560	HOXD12	2.210314138
79	97	215	OPHSV0400009701	-	2.080361843
80	91	230	OPHSV0400007331	-	2.341115519
81	94	224	H300021814	TTC21B	2.14183381
82	84	253	OPHSV0400007415	-	2.869227693
83	91	235	OPHSV0400000769	FPR1	2.393248452
84	97	221	OPHSV0400006196	PROM1	2.076048494
85	94	231	H200007593	LACE1	2.446628319
86	95	231	OPHSV0400012290	-	2.451701182
87	93	237	H200000957	C9orf58	2.560629813
88	99	225	OPHSV0400003696	Q6P168_HUMAN	2.192520584
89	86	260	OPHSV0400010183	-	3.190838823
90	93	243	OPHSV04TC000027	PSMD2	3.010452495
91	85	268	H200011078	SMAD7	2.961244321
92	98	236	OPHSV0400011877	-	2.382112973
93	102	227	H300001543	CCDC75	2.10796727
94	104	223	H300001191	OR5H1	2.019550725
95	105	230	OPHSV0400009844	-	2.101756744
96	94	257	H300019076	SLC17A3	2.653765221
97	115	213	H200002400	DUSP11	2.087971534
98	108	229	OPHSV0400008858	-	2.351475745
99	98	255	OPHSV0400011210	-	2.319808977
100	102	245	H300006590	Q8N9F6_HUMAN	2.161567647
101	105	240	OPHSV0400009390	RHBDF1	2.302786766
102	118	215	H300001076	-	2.160921945
103	107	238	OPHSV0400012194	-	2.446652694
104	112	228	OPHSV0400006153	-	2.145549222
105	87	296	OPHSV0400010611	-	2.561916424
106	107	243	OPHSV0400007822	Q6AI09_HUMAN	2.492703822
107	110	237	H200003415	EFS	2.252217021
108	111	236	OPHSV0400009266	-	2.142317147
109	105	258	OPHSV0400011216	-	2.41344609
110	101	269	H300010500	FAM78A	2.40660584
111	105	260	OPHSV0400004510	Q8N862_HUMAN	2.281303397
112	110	249	H200006614	-	2.316962305
113	94	293	OPHSV0400013129	-	2.644427673
114	96	289	OPHSV0400006576	ANGPT1	2.372620995
115	107	263	H200009600	C12orf46	2.254614185
116	113	250	H200008329	UTP20	2.201026777
117	111	256	OPHSV0400011442	-	2.283008987
118	111	257	OPHSV0400011218	-	2.236234731
119	117	244	H200005378	FAM114A1	2.166319984
120	107	268	H300020058	GTF2H1	2.637901907
121	113	254	X58149_50	-	2.341767028

122	104	278	OPHSV0400000877	MCF2L	2.750995194
123	106	276	H300013564	CRTAC1	2.408891657
124	106	276	OPHSV0400002891	C20orf186	2.403954935
125	106	279	OPHSV0400007673	-	2.230408888
126	108	275	H200000572	LCT	2.187676255
127	105	285	H200007529	ZNF256	2.73081293
128	106	286	H200017054	IGFBP6	2.414094406
129	96	321	H200016159	TCF19	2.763630789
130	96	323	OPHSV0400007474	HMX2	2.637407936
131	89	353	H200010863	KCNN2	3.328090489
132	103	307	H200014057	NUP93	2.596304719
133	111	286	H300019452	MARCH7	2.178372393
134	126	255	H200006637	PCMT1	2.146268997
135	121	268	OPHSV0400008373	-	2.202048632
136	116	281	OPHSV0400009760	Q8WZ26_HUMAN	2.033297154
137	108	302	H300018272	CAV3	2.433015372
138	134	245	H200006772	DPT	2.019738257
139	123	275	H200011808	-	2.204015441
140	88	390	OPHSV0400001025	FAM11B	3.019784808
141	115	305	OPHSV0400010666	-	2.592144277
142	124	288	H300005508	PKDREJ	2.277134845
143	126	288	OPHSV0400012849	-	2.107434725
144	119	305	OPHSV0400013120	-	2.427351104
145	120	306	OPHSV0400002187	KRBA2	2.168764108
146	123	299	H200008154	SERPINF1	2.194951035
147	121	308	OPHSV0400011415	-	2.476564405
148	138	272	H300011441	SERPINC1	2.057312387
149	140	269	H300010146	NP_001073958.1	2.000110335
150	134	284	ALIEN2_50	-	2.112981327
151	136	280	OPHSV0400002116	MYOM3	2.047612725
152	127	302	OPHSV0400001235	DEFB112	2.277065807
153	132	291	H200014908	-	2.055530623
154	124	315	H200017785	PLXNB3	2.694273794
155	140	280	OPHSV0400003470	BCAS3	2.127718419
156	114	348	OPHSV0400009267	-	2.432862404
157	123	324	H300016895	PDE4DIP	2.276811427
158	134	299	OPHSV0400011485	-	2.05427361
159	138	291	OPHSV0400008829	MSX2	2.183967764
160	127	318	OPHSV0400008394	-	2.579057054
161	131	310	OPHSV0400007036	-	2.249326641
162	119	348	OPHSV0400006317	KDELR3	3.084361353
163	118	351	H200003301	C9orf72	3.117247306
164	105	401	OPHSV0400005480	CRAMP1L	2.677695989
165	97	437	OPHSV0400011310	LCORL	3.630785183
166	112	381	OPHSV0400002270	TSSK2	2.835748202
167	127	337	H300022299	SPIRE2	2.462490529
168	108	401	OPHSV0400008374	-	3.016329737
169	138	328	OPHSV0400004810	NP_997386.1	2.067977301
170	142	320	OPHSV0400002582	DCP2	2.086062508
171	116	395	ATC04	-	3.081124087
172	140	328	OPHSV0400009174	-	2.191103509
173	113	411	M2NC000004	-	2.414977219
174	138	339	OPHSV0400000763	FCGR1A	2.023218574
175	138	339	OPHSV0400003368	-	2.030936491

176	124	379	OPHSV0400007574	-	2.51197884
177	140	340	OPHSV0400011411	KITLG	2.058995276
178	144	334	H200013755	KLK7	2.089856404
179	141	343	H300006383	NDST2	2.213312464
180	149	328	H300019038	AGBL3	2.094771995
181	112	441	OPHSV0400005037	ZNF524	3.490798316
182	119	420	OPHSV0400012845	-	2.797863994
183	145	348	OPHSV0400008791	Q68DG8_HUMAN	2.201231286
184	146	349	H300008545	-	2.055367498
185	141	363	OPHSV0400008059	-	2.146595509
186	137	377	OPHSV0400007821	-	2.055073863
187	132	393	OPHSV0400001730	CALN1	2.322364001
188	141	370	H300001106	-	2.197466711
189	133	401	H300019972	FBXW11	2.882102847
190	125	430	OPHSV0400008111	-	2.63589045
191	138	391	H300020742	DIP2A	2.327566705
192	139	393	H200014271	UBE2J1	2.414266459
193	145	381	OPHSV0400012179	-	2.249842265
194	140	395	H200011877	P2RXL1	2.430591141
195	146	396	OPHSV0400001465	-	2.276022932
196	145	403	OPHSV0400002912	-	2.406894703
197	131	447	H200003168	SSX2IP	2.444189252
198	109	540	H200011022	ERCC6	3.443527013
199	167	357	OPHSV0400010200	-	2.143688135
200	155	388	H200015940	USF1	2.277405303
201	166	368	H200010981	ZNF750	2.002509898
202	157	392	H200000567	BLK	2.008386949
203	137	450	OPHSV0400000046	TCFL5	2.425911889
204	139	449	H300015141	PHKA2	2.638506143
205	171	369	H300015003	MSLN	2.288131011
206	169	375	H200011893	CLPX	2.026606574
207	138	463	OPHSV0400000902	KLF14	2.899274881
208	176	365	OPHSV0400010960	-	2.172651502
209	145	445	OPHSV0400010221	C10orf25	2.779741761
210	158	409	H200000972	SRGAP2	2.344763496
211	163	397	H200003517	IRX5	2.116901732
212	146	450	OPHSV0400013419	-	2.069381648
213	164	411	H300012021	USP44	2.027568529
214	135	500	M2NC000005	-	2.696912565
215	126	538	H300005877	SLC3A1	3.290146345
216	170	402	H300020077	DNM1L	2.588754112
217	173	399	H300008362	-	2.111619854
218	162	428	OPHSV0400012256	-	2.461962182
219	154	462	OPHSV0400000836	PURG	2.33677741
220	166	430	H200015703	KCNK6	2.12948615
221	140	510	OPHSV0400011046	-	2.874661776
222	168	430	OPHSV0400001395	MAML3	2.019851437
223	173	421	H300020584	USP25	2.034107131
224	173	421	OPHSV0400006522	-	2.066686273
225	135	553	OPHSV0400012941	-	3.306850641
226	177	430	OPHSV0400012465	-	2.029815014
227	171	449	OPHSV0400011244	-	2.216272793
228	168	460	OPHSV0400007939	-	2.380908097
229	170	455	OPHSV0400012770	-	2.38656991

230	183	424	OPHSV0400007458	-	2.010293655
231	163	487	H200005947	GJA1	2.181716543
232	178	450	OPHSV0400013216	-	2.060392776
233	170	472	OPHSV0400000534	ZNF782	2.137392238
234	157	512	H300020108	ANKRD10	2.421815566
235	176	460	OPHSV0400002852	KRTAP22-1	2.181345851
236	154	528	H200011985	MANBA	2.502683743
237	181	451	H200006451	NFATC4	2.028450211
238	176	464	H200007362	CCDC19	2.152529099
239	162	513	OPHSV0400010385	-	2.494589781
240	184	457	H300011218	-	2.2324185
241	172	498	H200014159	PARD6G	2.143457255
242	170	505	H300018294	VNN2	2.282405548
243	186	462	OPHSV0400013227	-	2.187596535
244	173	509	H200003235	ANAPC2	2.166495152
245	163	542	OPHSV0400012841	-	2.385048338
246	198	450	OPHSV0400005508	TP53AP1	2.169070565
247	140	644	H200008589	ATP6V1C2	2.763744425
248	155	589	OPHSV0400002581	Q5BKX7_HUMAN	3.128566103
249	145	636	H300003382	OR52K2	2.517745994
250	179	520	OPHSV0400004142	XRRA1	2.190268957
251	190	492	OPHSV0400006279	ACSBG1	2.370273233
252	164	575	OPHSV0400002410	hCG_2040376	2.065798995
253	153	617	OPHSV0400010042	-	2.415064075
254	168	562	H300020430	PLCB4	2.080548351
255	198	481	H200000384	SERPIND1	2.11376884
256	130	748	OPHSV0400012700	-	3.033266325
257	172	566	OPHSV0400010645	-	2.087449553
258	189	518	OPHSV0400002565	PDE8B	2.240848432
259	189	523	H200018234	-	2.300273925
260	178	560	OPHSV0400008017	-	2.190202125
261	183	548	H300007315	SLC6A1	2.269113144
262	201	501	H200001480	MRPL27	2.134342642
263	161	636	OPHSV0400013243	-	2.482052403
264	158	667	OPHSV0400000988	-	3.006110418
265	175	604	OPHSV0400009684	-	2.358778901
266	184	583	OPHSV0400011767	-	2.349951207
267	198	542	H200002403	NAT8B	2.025258314
268	199	544	H200017552	RBX1	2.037581771
269	170	657	H200014979	LCN2	2.938385965
270	189	595	OPHSV0400006139_80	-	2.002224736
271	202	564	OPHSV0400002467	TBX1	2.008197168
272	154	761	OPHSV0400006754	MLL3	2.728935076
273	179	656	H300019985	STK3	2.193144691
274	185	640	OPHSV0400001788	MYO7B	2.300900788
275	182	661	OPHSV0400002955	KIAA0562	2.539009734
276	195	618	OPHSV0400009056	-	2.5529745
277	190	647	H200012407	CASP8AP2	2.361488322
278	207	599	H200005458	TFAP2C	2.058240867
279	206	607	OPHSV0400011805	-	2.015068386
280	219	580	H300009838	IL1RAPL1	2.09579337
281	182	706	OPHSV0400008955	-	2.591116592
282	213	605	H200008777	GUCA1B	2.008123221
283	194	667	H200015408	KIAA0953	2.453255918

284	225	584	H300022389	ATXN1	2.108222134
285	202	661	H300008010	Q8NGD7_HUMAN	2.053681066
286	178	757	OPHSV0400011168	-	2.561616303
287	214	639	OPHSV0400000374	ZNF740	2.221110327
288	232	596	H200006802	RASSF2	2.309207152
289	234	591	OPHSV0400010640	-	2.188102957
290	222	627	H200001239	MON1A	2.31819547
291	250	561	H300016040	PKNOX1	2.054014926
292	239	594	H300018631	ADRBK2	2.253300271
293	250	568	H200005627	TAF15	2.054449292
294	229	621	H200017980	Q99946-2	2.345469748
295	233	614	OPHSV0400012469	-	2.164154977
296	243	590	H200013269	PGLYRP1	2.096551613
297	219	660	OPHSV0400002687	EFHC2	2.192099489
298	215	673	OPHSV0400006123	TNKS	2.084183897
299	208	696	OPHSV0400009261	-	2.040224292
300	195	746	OPHSV0400004054	-	2.311769704
301	206	707	OPHSV0400009787	-	2.303921437
302	216	678	OPHSV0400003228	RGPD3	2.323192203
303	200	738	H200005745	SPG21	2.457534263
304	195	758	OPHSV0400008978	NP_001013737.1	2.764059696
305	245	610	H200016045	HOXA3	2.156943171
306	233	645	OPHSV0400006699	-	2.021703489
307	231	652	H200016325	CTSZ	2.032580475
308	210	725	OPHSV0400009789	-	2.121389824
309	210	728	OPHSV0400011248	-	2.117821155
310	223	690	H200006412	ITPR3	2.123314612
311	241	642	H300021401	SNTG2	2.253546871
312	249	622	H300008106	Q96N33_HUMAN	2.081957906
313	216	722	OPHSV0400009278	-	2.055041281
314	193	821	OPHSV0400000344	MAP3K1	3.062581873
315	224	714	OPHSV0400000653	PHYHIPL	2.511443042
316	250	656	H200006784	REPS2	2.123862857
317	220	747	H300005750	HSPA5	2.3992712
318	221	752	OPHSV0400000611	RYK	2.280313921
319	227	739	H200007996	DHX32	2.345290992
320	243	693	OPHSV0400010838	-	2.143119676
321	246	689	H300007676	NAPSB	2.012674027
322	212	809	H300021954	-	2.312251883
323	230	759	H300020737	C21orf2	2.283313893
324	232	764	H200001744	ABHD4	2.101327371
325	247	724	H200017494	PHF11	2.030011541
326	252	718	H300008195	Q8N2W8_HUMAN	2.13018308
327	256	713	H300016451	C7orf26	2.582679749
328	217	849	H200004207	SEMA3F	2.494578657
329	256	724	OPHSV0400009201	-	2.062185956
330	227	821	H300009007	PRAMEF10	2.471860284
331	251	749	OPHSV04TC000047	-	2.498555479
332	249	763	OPHSV0400008420	-	2.12693199
333	256	755	OPHSV0400011825	-	2.09690656
334	238	820	H200015606	IL7R	2.228679392
335	265	741	H200019539	NP_115991.1	2.039647079
336	296	668	H300001919	CCDC107	2.012194357
337	234	855	H300017668	-	2.159188584

338	231	869	H300003104	SUSD2	2.094879786
339	200	1020	OPHSV0400007096	ZNF321	2.832292984
340	249	820	OPHSV0400002222	NP_689993.1	2.047631338
341	244	841	OPHSV0400009590	-	2.753948839
342	232	889	OPHSV0400000971	EIF4H	2.63414328
343	272	760	OPHSV0400002303	NDN	2.023959499
344	259	804	H300011601	USP7	2.349485939
345	257	866	OPHSV0400007766	TRIM61	2.02118063
346	270	831	OPHSV0400004937	-	2.128651718
347	260	863	OPHSV0400009689	-	2.244578685
348	271	828	H300016213	ABCA12	2.109995854
349	253	899	OPHSV0400010852	-	2.107274147
350	240	960	H300014063	MYB	2.083788793
351	247	934	OPHSV0400006858	-	2.027782346
352	295	790	OPHSV0400007992	-	2.059463884
353	341	703	OPHSV04TC000025	PSMD2	2.102157437
354	316	764	OPHSV0400011400	-	2.116851945
355	291	833	H300022270	ZNF294	2.139793321
356	305	797	H200004499	ALG2	2.001478278
357	216	1146	OPHSV0400011739	-	3.033019536
358	283	881	H300014222	KIAA1602	2.547081608
359	213	1192	OPHSV0400009912	-	2.470700408
360	268	987	OPHSV0400002530	IZUMO1	2.087508204
361	215	1259	H200002018	CCDC113	2.875452463
362	262	1089	OPHSV0400008644	-	2.259275797
363	288	1011	OPHSV0400011968	-	2.153692127
364	325	915	OPHSV0400003825	LHX8	2.171877508
365	345	873	OPHSV0400007138	-	2.03536024
366	343	885	H200009951	JMJD2B	2.11864925
367	323	940	OPHSV0400013348	hsa-mir-24-1	2.297555409
368	318	969	H200015537	RECQL	2.140628447
369	321	960	OPHSV0400010161	-	2.090495993
370	286	1089	OPHSV0400011713	-	2.209915293
371	260	1225	OPHSV0400006570	-	2.493260814
372	281	1139	H200014208	VNN3	2.30658056
373	344	939	H300021857	ELL3	2.031194697
374	335	966	OPHSV0400011548	SNAG1	2.069582411
375	316	1027	H300002800	AHNAK	2.216700044
376	323	1013	OPHSV0400002174	C9orf66	2.222867263
377	344	958	OPHSV0400012308	-	2.049023565
378	293	1141	H300009173	CITED4	2.652620271
379	336	996	OPHSV0400007441	-	2.201469073
380	331	1017	H200012814	Q8NBX4_HUMAN	2.271967205
381	276	1263	H200017696	PCDHA13	2.427152077
382	374	950	H200010682	-	2.001155605
383	324	1120	H300016150	EEF1B2	2.67326859
384	346	1056	OPHSV0400006249	-	2.25870472
385	267	1380	H200000432	CYP4A11	2.505645217
386	319	1175	H300004990	NPAS4	2.026568632
387	267	1410	OPHSV0400012726	-	2.356420144
388	282	1336	AF159801_70	-	2.26027997
389	325	1194	OPHSV0400012885	-	2.171952524
390	300	1300	H300020299	Q96CK5_HUMAN	2.24887773
391	244	1602	ALIEN6_90	-	3.012581037

392	383	1060	H200003420	SAMSN1	2.068015042
393	341	1201	OPHSV0400001801	SETD3	2.333042272
394	279	1514	OPHSV0400003294	NP_997198.2	2.503417559
395	342	1248	OPHSV0400008697	-	2.181131238
396	335	1291	OPHSV0400010537	-	2.280537787
397	332	1339	OPHSV0400013296	hsa-mir-128a	2.437403564
398	438	1029	H200007266	LHX1	2.018362319
399	295	1606	OPHSV0400013229	-	2.65037451
400	244	2056	H300000715	Q9UI77_HUMAN	3.806280563
401	374	1358	H200008080	ARMCX3	2.540881289
402	394	1327	H300017730	TJP2	2.246750458
403	385	1360	H300021927	MAK10	2.300529445
404	374	1427	OPHSV0400005989	NP_001004318.1	2.065932554
405	364	1469	OPHSV0400001371	ACOT4	2.065825476
406	353	1537	H300022125	VPS33A	2.444366632
407	372	1470	OPHSV0400012166	CENPP	2.345837873
408	397	1453	OPHSV0400006353	NUSAP1	2.360193549
409	435	1338	OPHSV0400004103	ALG1	2.084094161
410	420	1387	OPHSV0400006397	ZNF8	2.064619657
411	344	1716	OPHSV0400010160	-	2.132742226
412	417	1459	OPHSV0400000023	-	2.633471358
413	390	1575	H200008552	CC2D1A	2.300689587
414	388	1598	OPHSV0400009591	-	2.387597551
415	506	1237	H200014745	SUSD5	2.182117028
416	342	1853	H200002292	NP_056996.2	2.333670966
417	384	1674	OPHSV0400003252	DCHS2	2.0574096
418	416	1570	H300020907	-	2.513978642
419	412	1600	OPHSV0400000542	-	2.46477044
420	431	1530	OPHSV0400003148	FAM124B	2.347888568
421	418	1623	OPHSV0400011315	-	2.22146964
422	410	1685	OPHSV0400004139	PCDH18	2.008006969
423	421	1648	OPHSV0400002454	NP_056274.3	2.005306931
424	455	1582	H300004433	UBQLNL	2.129904307
425	470	1532	OPHSV0400013310	hsa-mir-143	2.218293015
426	428	1698	H200004042	ARRDC2	2.32415642
427	434	1704	H200004973	INTS2	2.131705837
428	392	1943	OPHSV0400008729	NP_001005751.1	2.898795325
429	480	1603	OPHSV0400009860	-	2.253567946
430	456	1714	H200002849	C1orf35	2.378780358
431	381	2066	H300018884	ARD1_HUMAN	2.646160375
432	402	1984	H200002485	DEF6	2.716351627
433	499	1610	H200008285	ZSCAN5	2.042498502
434	484	1667	OPHSV0400010147	-	2.136726695
435	498	1670	H300021309	ZNF507	2.167617925
436	458	1827	OPHSV0400005826	CSAG3B	2.037598345
437	466	1840	H200003502	CENTD3	2.152245212
438	487	1764	OPHSV0400008722	LCN8	2.078142086
439	492	1768	OPHSV0400006622	DMRTC1	2.02808532
440	421	2069	H300006583	SLC9A10	2.530455087
441	494	1943	H300022635	LOC440776	2.267157817
442	481	2005	OPHSV0400004480	GPBP1L1	2.524277543
443	484	2040	OPHSV0400000471	ADAM8	2.094921579
444	508	2004	H300019864	MARK1	2.027751519
445	411	2658	OPHSV0400008701	-	2.798777466

446	501	2282	H300017501	CD74	2.230257215
447	500	2297	OPHSV0400013074	-	2.182446
448	501	2297	H200017407	ANGPT4	2.326597574
449	533	2198	OPHSV0400009205	-	2.293621691
450	519	2340	H300003340	TIMM10	2.072582757
451	464	2636	OPHSV0400002576	Q9P1M3_HUMAN	2.330043119
452	421	3000	H200004752	PPP1R16B	2.844028585
453	597	2163	H300009043	Q8NAN8_HUMAN	2.293534924
454	517	2533	OPHSV0400000310	GABRA3	2.638099023
455	633	2085	H300016715	C4orf18	2.093126925
456	612	2190	H300014606	-	2.073082382
457	586	2374	OPHSV0400010799	-	2.057024685
458	522	2785	OPHSV0400002624	UBE1L	2.526108598
459	633	2303	OPHSV0400013422	-	2.042942578
460	802	2069	OPHSV0400006583	THAP6	2.004685805
461	494	3425	H300018839	ISCU	3.000587084
462	559	3131	OPHSV0400008730	-	2.671611174
463	597	3090	H200004603	TAF13	2.456133835
464	665	2904	H300001954	NOB1	2.139440796
465	568	3445	H300021145	ANGPTL4	2.329998463
466	610	3262	H300022218	NP_060223.2	2.350125001
467	713	2840	H200004583	GPAM	2.172537953
468	798	2700	OPHSV0400010922	-	2.110787017
469	789	2895	H300019644	SIRT5	2.022871047
470	773	2958	OPHSV0400001351	RPRM	2.026662102
471	659	3568	OPHSV0400011242	Q71RF5_HUMAN	2.968339001
472	785	3097	OPHSV0400002229	C21orf129	2.1083706
473	660	3753	H200000022	GABPA	2.390951183
474	822	3070	OPHSV0400013297	hsa-mir-128b	2.056659158
475	837	3171	H200012396	MRPS27	2.082132252
476	722	3960	H200016720	KIF23	2.488384509
477	783	3675	OPHSV0400008648	-	2.273414217
478	666	4579	H300018522	MAP2K5	2.231742619
479	790	3910	H300013740	Q3KNT7-3	2.045595645
480	711	4446	H200013986	MTHFD2	2.230191597
481	934	3775	H200011818	PSME4	2.022910997
482	1002	3688	OPHSV0400011008	SNX12	2.081326253
483	1008	3738	X14212	-	2.207016903
484	969	3925	OPHSV0400010090	PHKA2	2.363479575
485	1144	3456	OPHSV0400006905	-	2.176716781
486	975	4531	OPHSV0400011840	-	2.043308344
487	925	4962	H300021503	ARSJ	2.089363688
488	908.8	5215.2	OPHSV0400013392	-	2.386609216
489	1127	4299	OPHSV0400010937	-	2.117202165
490	1247	5230	H300006821	NP_001025045.1	2.130235017
491	1474	4695	OPHSV0400011489	-	2.055507666
492	1369	5362	H200013363	ZNF544	2.078317894
493	1313	5773	OPHSV0400005653	DNAH2	2.007514974
494	1378	5720	H300006097	CCL17	2.134272803
495	1095	7417	OPHSV0400000970	FAM20C	2.435515265
496	1390	6426	OPHSV0400011606	-	2.030954329
497	1465	6817	OPHSV0400001008	NP_060219.2	2.045490922
498	1662	6871	OPHSV0400010639	-	2.14281325
499	1851	6481	OPHSV0400002684	-	2.217185624

500	1888	7056	H200002409	ACSS2	2.17511562
501	1841	7658	H300011550	MYOCD	2.087354447
502	1893	7560	OPHSV0400007617	IKZF1	2.039419026
503	1845	8850	OPHSV0400004479	-	2.160244776
504	2056	8114	H200011064	OGT	2.107303849
505	2023	10449	OPHSV0400006666	NP_001072995.1	2.512892679
506	2412	8854	OPHSV0400005502	-	2.02041322
507	2420	9352	H300010020	GSTA3	2.113136191
508	2467	11832	H200012360	-	2.010497084
509	2896	13382	OPHSV0400006561	NP_612412.2	2.10756976
510	3099	13074	H300021673	YBX1	2.035778287
511	3772	12384	OPHSV0400007078	-	2.101036276
512	3643	14642	OPHSV0400012406	-	2.018686105
513	4166	13340	H200003429	ARRDC3	2.066966167
514	3916	14934	H200006580	GABPB2	2.232552193
515	4023	14636	OPHSV0400012823	-	2.239157219
516	3429	18355	H200001437	MCAT	2.38305364
517	3741	21039	OPHSV0400006736	-	2.423557626
518	5149	20532	H300019342	ACTR3B	2.252596234
519	6660	23779	H300015824	-	2.055153798
520	9425	29250	H200001745	UBADC1	2.135201495
521	9253	29916	H300008997	VAMP4	2.149958907
522	12417	36946	H300007847	-	2.123132989
523	17015	39740	OPHSV0400006285	NELFB_HUMAN	2.098158565
524	18975	46894	H200018769	ATP5H	2.145358485
525	25928	48110	H200002757	AIFM1	2.192749174
526	32024	55806	H300009305	UBE2U	2.342526139

Downregulated genes

	Cy3	Cy5	Id	Symbol	Zscore
1	197	121	OPHSV0400007456	-	-2.11081
2	210	121	OPHSV0400006848	-	-2.01113
3	234	119	H300001568	Q9NRE4_HUMAN	-2.07815
4	255	119	H200019503	CKM	-2.1437
5	257	120	OPHSV0400001247	Q96NJ7_HUMAN	-2.07167
6	261	122	OPHSV0400012252	-	-2.04285
7	258	125	OPHSV0400007775	-	-2.04007
8	255	128	OPHSV0400000116	SLC7A14	-2.09736
9	277	125	OPHSV0400004456	Q6W349_HUMAN	-2.10307
10	291	119	OPHSV0400004255	-	-2.35666
11	284	123	OPHSV0400009794	-	-2.68144
12	277	127	OPHSV0400005814	ENSA	-2.35878
13	278	130	OPHSV0400005934	NP_001001682.1	-2.02837
14	282	129	OPHSV0400004123	LOC644334	-2.12314
15	306	121	H200016629	PLCG1	-2.11439
16	286	132	OPHSV0400007897	Q6ZU01_HUMAN	-2.00373
17	317	122	H200014047	MUT	-2.3836
18	303	135	OPHSV0400008189	RPL18	-2.08218
19	321	128	H200003971	PARP8	-2.29418
20	321	131	OPHSV0400000484	-	-2.0123

21	318	137	H200017324	GALNT3	-2.03712
22	325	137	OPHSV0400003563	RHBDD1	-2.30427
23	289	155	H200014586	IL10	-2.02675
24	359	132	H300019027	ZC3H7A	-2.23619
25	378	126	H200000405	DCLRE1A	-2.63408
26	399	121	H200012668	ZNRF2	-2.8518
27	353	139	OPHSV0400003486	-	-2.0572
28	334	148	H200009646	SIAH1	-2.0464
29	367	138	H300005894	TAAR1	-2.1784
30	346	147	H300006447	MT1G	-2.02139
31	356	143	OPHSV0400010589	-	-2.0865
32	358	146	OPHSV0400000951	Q8N9C5_HUMAN	-2.03061
33	383	141	H200011023	GABRR2	-2.01623
34	369	148	H300003648	C1orf122	-2.19417
35	401	137	H300013839	METT5D1	-2.72528
36	370	150	OPHSV0400000073	RAB3D	-2.26636
37	401	141	H200006883	ITGB3BP	-2.11788
38	401	141	OPHSV0400003869	LYPD4	-2.0972
39	411	142	OPHSV0400005222	Q6ZS35_HUMAN	-2.60042
40	415	144	OPHSV0400006678	VN1R5	-2.3444
41	404	151	OPHSV0400012067	-	-2.13117
42	496	124	H300003993	C10orf49	-2.49348
43	361	174	OPHSV0400013105	-	-2.05216
44	420	151	H300002753	Q8NAM0_HUMAN	-2.8218
45	450	145	OPHSV0400003574	ZDHHC9	-2.15723
46	430	152	H200008351	TMCC1	-2.11192
47	412	161	OPHSV0400005570	Q6NXN2_HUMAN	-2.05716
48	446	152	H200005503	C13orf33	-2.4318
49	418	167	OPHSV0400010604	-	-2.30811
50	399	176	H200002676	NGFRAP1	-2.06586
51	396	178	H300004905	-	-2.00019
52	514	140	OPHSV0400004331	RFPL1	-2.85123
53	438	169	H200000048	GLP1R	-2.39595
54	454	168	OPHSV0400010811	-	-2.06932
55	443	178	H200005282	FAAH2	-2.19123
56	420	189	OPHSV0400012146	-	-2.1143
57	448	180	H200006242	USP14	-2.0872
58	454	184	OPHSV0400010894	-	-2.03231
59	465	181	H200007199	SMPX	-2.06006
60	507	167	OPHSV0400005279	-	-2.12103
61	464	185	OPHSV0400002837	Q86VD6_HUMAN	-2.00497
62	473	182	H200000542	TRAF1	-2.395
63	588	147	OPHSV0400005030	-	-3.21004
64	513	173	OPHSV0400002065	Q86YU6_HUMAN	-2.20566
65	483	185	H200011013	MAS1	-2.20096
66	504	180	H200004984	WDR53	-2.02149
67	538	176	H300007745	OR2G3	-2.0953
68	526	185	OPHSV0400004527	-	-2.00191
69	600	171	H200011642	ATP10B	-2.30559
70	542	190	H300022210	NXF2	-2.02432
71	582	192	H200008916	MED12L	-2.29486
72	632	181	H300017225	HERC4	-2.20189
73	686	172	H300021436	Q7Z4R2_HUMAN	-2.03373
74	587	213	OPHSV0400011574	-	-2.1066

75	580	217	H300010407	-	-2.38451
76	636	214	OPHSV0400010758	-	-2.12211
77	604	238	H300019554	IPO11	-2.05726
78	687	215	OPHSV0400006826	-	-2.20023
79	746	229	OPHSV0400004923	-	-2.31008
80	707	250	H300014773	RANBP2	-2.01202
81	841	219	OPHSV0400005044	Q6PID2_HUMAN	-2.41906
82	818	235	OPHSV0400012111	BMPR2	-2.05341
83	793	261	OPHSV0400010983	THRAP3	-2.07299
84	810	256	OPHSV0400005296	Q9NSI7_HUMAN	-2.08478
85	898	240	OPHSV0400004973	-	-2.63391
86	781	279	OPHSV0400004053	RAD51L1	-2.01211
87	911	241	OPHSV0400004834	-	-2.12492
88	910	245	OPHSV0400004947	Q6PID2_HUMAN	-2.38848
89	960	239	H200009381	OPA3	-2.37172
90	1095	233	OPHSV0400004939	-	-2.21921
91	952	277	OPHSV0400005681	CASC2	-2.32931
92	900	302	OPHSV0400008444	-	-2.25272
93	858	324	OPHSV0400012135	-	-2.11368
94	869	322	H300015140	FBXL18	-2.19455
95	871	330	H300009200	-	-2.18092
96	923	322	H300020874	SGOL2	-2.0497
97	1110	274	OPHSV0400001811	Q5T2Q4_HUMAN	-2.24275
98	1073	290	H300003366	STX19	-2.06348
99	961	352	H300017054	-	-2.03331
100	1204	285	OPHSV0400001703	CLRN1	-2.09561
101	1040	334	OPHSV0400001350	-	-2.06013
102	934	377	H200008692	CCDC129	-2.11615
103	926	390	OPHSV0400001759	Q6ZSY1_HUMAN	-2.02308
104	1067	341	H200010381	INSR	-2.14895
105	1114	341	OPHSV0400004647	Q6ZNC3_HUMAN	-2.08953
106	1322	319	OPHSV0400009807	-	-2.52125
107	1128	374	OPHSV0400004615	Q6ZNC3_HUMAN	-2.00149
108	1188	359	H300010114	SFT2D1	-2.23768
109	1380	322	OPHSV0400004316	Q6ZU70_HUMAN	-2.52067
110	1301	352	H300011229	NP_001073999.1	-2.15214
111	1570	340	H300001051	Q8N7N2_HUMAN	-2.36375
112	1726	311	OPHSV0400005331	-	-2.86498
113	1593	349	OPHSV0400009801	-	-2.49363
114	1435	402	OPHSV0400004716	LIN1_HUMAN	-2.22572
115	1626	361	OPHSV0400005647	-	-2.36932
116	1648	360	OPHSV0400009812	-	-2.45917
117	1483	403	OPHSV0400004299	Q68DE7_HUMAN	-2.02414
118	1521	417	OPHSV0400011012	-	-2.06005
119	1792	373	OPHSV0400009796	-	-2.68712
120	1559	432	OPHSV0400004550	-	-2.20136
121	1559	438	H300014104	-	-2.05546
122	1849	403	OPHSV0400009814	-	-2.33559
123	1686	450	H300019541	TMPO	-2.1459
124	1704	452	OPHSV0400004418	NP_079198.2	-2.10021
125	1942	413	H300003717	NNT	-2.16505
126	1622	538	H200017579	TMEM68	-2.05119
127	1859	479	OPHSV0400008418	-	-2.06945
128	1788	503	OPHSV0400000790	CD2BP2	-2.03314

129	1894	488	H300000825	Q6ZUR4_HUMAN	-2.00639
130	1872	550	H200014948	KCNK2	-2.03628
131	2081	500	OPHSV0400009805	-	-2.1336
132	2108	501	OPHSV0400004701	Q6ZND9_HUMAN	-2.16624
133	2219	486	OPHSV0400009799	-	-2.39337
134	1881	595	H300006004	RAB36	-2.12209
135	2125	543	H200013667	CYB561D2	-2.0664
136	2524	548	OPHSV0400005578	-	-2.05802
137	2473	562	OPHSV0400005176	-	-2.08606
138	2437	598	H200014461	PABPC5	-2.14602
139	2598	573	OPHSV0400005359	Q6ZUH1_HUMAN	-2.15572
140	2365	634	OPHSV0400004513	LIN1_HUMAN	-2.00422
141	2820	538	OPHSV0400008877	-	-2.70965
142	2435	627	OPHSV0400005716	-	-2.18098
143	2281	704	OPHSV0400004967	-	-2.30009
144	2604	657	OPHSV0400005420	ODF2L	-2.04491
145	3271	551	OPHSV0400009797	-	-2.4669
146	2438	753	OPHSV0400004662	-	-2.14265
147	2500	755	H300010439	RMND1	-2.38424
148	2897	704	OPHSV0400005827	MT-CO1	-2.30132
149	2643	774	H300003806	Q8NBL2_HUMAN	-2.10924
150	3044	734	OPHSV0400006578	TBC1D20	-2.06846
151	3526	651	OPHSV0400009806	-	-2.35278
152	3198	726	OPHSV0400005061	LIN1_HUMAN	-2.00293
153	3032	779	OPHSV0400004581	Q6ZNC3_HUMAN	-2.17947
154	2868	841	H300010305	ADAMTSL4	-2.22061
155	2892	846	H300015880	USP19	-2.08204
156	3249	755	H300009729	-	-2.37139
157	3024	820	OPHSV0400005569	-	-2.38798
158	3029	829	H200014678	O94914_HUMAN	-2.3073
159	3235	797	H300011879	-	-2.22352
160	3633	736	OPHSV0400004784	ODF2L	-2.10962
161	3519	790	OPHSV0400004483	Q6PID2_HUMAN	-2.01332
162	3802	734	OPHSV0400005004	LIN1_HUMAN	-2.267
163	2916	969	OPHSV0400008262	-	-2.05435
164	2972	956	OPHSV0400000452	TRIM64	-2.20559
165	2930	990	H300012759	UNG2	-2.09731
166	3094	1027	OPHSV0400011361	-	-2.06756
167	3384	948	OPHSV0400003111	Q6ZTS9_HUMAN	-2.1338
168	3890	842	OPHSV0400005094	Q6PID2_HUMAN	-2.11348
169	3793	866	OPHSV0400005225	ODF2L	-2.02869
170	3706	903	H300006616	OR7G2	-2.44278
171	3809	953	H200013812	C4orf16	-2.07201
172	3989	1008	H300009443	ZDHHC20	-2.06275
173	5276	860	OPHSV0400009798	-	-2.54502
174	4675	1047	OPHSV0400009802	-	-2.04327
175	5019	1064	H300011483	SAKS1_HUMAN	-2.0629
176	4749	1135	H300003339	OR4C46	-2.26809
177	4237	1288	H200000308	SLC6A6	-2.01375
178	4316	1280	H200011338	SERPINB9	-2.16325
179	4057	1371	H300013631	WDR7	-2.04475
180	5123	1166	H300012524	-	-2.50205
181	5381	1126	OPHSV0400009813	-	-2.10092
182	5130	1315	OPHSV0400005855	GPX6	-2.08718

183	5401	1378	H200013976	SETD1B	-2.16347
184	5450	1563	OPHSV0400003983	-	-2.07178
185	5753	1558	H300004038	OR2AG1	-2.26293
186	5754	1591	OPHSV0400007302	-	-2.33715
187	5594	1678	H300005926	JPH1	-2.08767
188	6291	1518	H200007051	ACTN2	-2.35264
189	5826	1653	OPHSV0400010572	-	-2.08045
190	6367	1700	H300013847	CRHR1	-2.15673
191	6912	1679	OPHSV0400005312	-	-2.14273
192	6878	1720	H300004494	OR5M9	-2.10331
193	7615	1714	H300007222	-	-2.17884
194	7569	1793	OPHSV0400004880	Q6ZUR4_HUMAN	-2.13867
195	7704	1773	OPHSV0400013194	ACOT11	-2.05908
196	7308	1950	H300000517	ARSK	-2.00235
197	7602	1919	H300016310	C20orf24	-2.0275
198	8193	1833	H300012133	MS4A3	-2.19486
199	7852	1918	H300004629	KRTAP9-3	-2.16483
200	7880	1960	H200001657	DUS2L	-2.28375
201	7587	2038	H300011152	ACE	-2.13952
202	8054	2109	OPHSV0400011112	-	-2.5108
203	8032	2126	OPHSV0400001825	Q8IZM0_HUMAN	-2.4755
204	7543	2283	H300014602	IL1R1	-2.06563
205	7412	2358	OPHSV0400009905	-	-2.11234
206	8637	2069	H200015123	PIK3R2	-2.64872
207	8241	2190	H200014206	CRABP2	-2.21734
208	8917	2057	H300017863	AGPAT4	-2.15806
209	9345	1980	H200012193	ST13	-2.39514
210	9882	1929	H200008074	-	-2.08702
211	9348	2107	OPHSV0400005558	GAS2L1	-2.10942
212	8785	2268	H300004605	NP_057574.2	-2.26747
213	8122	2499	OPHSV0400005482	NANOG	-2.08416
214	8500	2421	OPHSV0400005376	-	-2.09823
215	9464	2230	H300005015	Q8NGY4_HUMAN	-2.32034
216	10485	2094	H300017851	-	-2.39572
217	10576	2076	OPHSV0400011094	-	-2.4252
218	8056	2832	H300008722	NP_001018022.1	-2.12409
219	10086	2291	H200015769	-	-2.51786
220	9800	2383	H200000503	CCNF	-2.37475
221	11273	2178	H200006031	PKIA	-2.15567
222	10226	2449	H200018241	SLC19A3	-2.05767
223	10748	2424	OPHSV0400008803	-	-2.2103
224	12620	2204	H300014942	PTPLA	-2.14605
225	12714	2294	H300021296	LBH	-3.02197
226	11516	2907	OPHSV0400005362	-	-2.25413
227	12852	2737	H200006356	DNASE1L1	-2.03423
228	13994	2750	OPHSV0400005643	-	-2.27129
229	12075	3277	H300017621	HTR4	-2.00026
230	13170	3032	H300013779	SRPK1	-2.11559
231	12349	3304	H200017294	GNPDA1	-2.01889
232	12360	3326	H200003731	RTCD1	-2.06945
233	15474	2838	H300008872	NP_001073961.1	-2.11163
234	13635	3583	H300011203	-	-2.59834
235	13527	3753	OPHSV0400004626	MPP7	-2.42981
236	15440	3345	OPHSV0400004348	Q86XG0_HUMAN	-2.37558

237	16222	3427	H300007470	OR10G4	-2.41515
238	15182	3720	OPHSV0400006410	ZNF440	-2.29634
239	12793	4559	H300005147	Q9BYB2_HUMAN	-2.01192
240	15083	3913	H300007061	S100PBP	-2.56184
241	14125	4281	H200016714	-	-2.00722
242	14506	4233	OPHSV0400003518	RBPSUH	-2.04847
243	14874	4157	OPHSV0400005544	UBE1	-2.10801
244	16561	3931	H300002888	MDM4	-2.01566
245	16543	3981	H200019038	ATG9A	-2.01048
246	17124	3880	OPHSV0400005582	-	-2.00725
247	14876	4727	H300001342	CBL	-2.05235
248	18837	3979	H300009440	PDIA6	-2.23311
249	18389	4105	H300008548	MFSD3	-2.1202
250	20416	3905	OPHSV0400005702	SVOP	-2.36632
251	19084	4205	H300016854	KIAA0310	-2.15421
252	20647	4205	OPHSV0400004672	LRRIQ2	-2.73418
253	18305	4769	OPHSV0400005555	-	-2.11984
254	20549	4451	H300007892	C4orf26	-2.05173
255	21588	4306	H200008625	ZNF136	-2.30054
256	22646	4162	H200011192	WDR40A	-2.23964
257	19681	4935	H300012496	-	-2.11475
258	21039	4712	H200011780	SLC30A3	-2.32067
259	22712	4456	H300018259	SSX6	-2.12231
260	22594	4504	H200001570	RASL11B	-2.16705
261	23057	4802	H300010656	DOT1L	-2.30154
262	22026	5041	OPHSV0400004551	-	-2.11028
263	22288	5009	H300008732	SIRPB1	-2.11355
264	19714	5757	H200014939	EDG5	-2.09092
265	21138	5738	H300014772	ZNF667	-2.17417
266	21825	5564	H300011278	AGPAT7	-2.09211
267	25135	4994	OPHSV0400005017	UBOX5	-2.29669
268	23409	5422	H200014281	CAMK1	-2.13791
269	21712	6166	H300007693	ATP6V0D2	-2.75325
270	25197	5423	H300013003	SLC25A1	-3.07505
271	25314	5612	H200007719	POR	-3.04637
272	28125	5150	H300006321	Q8N9C2_HUMAN	-3.07675
273	27375	5466	H300006510	Q00849_HUMAN	-2.7833
274	24928	6011	H200010210	C1orf113	-2.77775
275	25780	5867	H300005428	SEC11B	-2.88209
276	25700	6145	OPHSV0400004886	NT5DC3	-2.57281
277	25013	6384	H300003389	-	-2.09076
278	32415	4933	H300007788	Q9UI23_HUMAN	-2.88303
279	26051	6282	H300007221	-	-2.06617
280	28113	5984	H300018057	PTGER3	-2.04572
281	27626	6284	H300014926	SARDH	-2.05283
282	28969	6075	H300007376	CHMP5	-2.30126
283	28503	6244	H300007179	-	-2.32732
284	24480	7631	OPHSV0400005514	Q6YL35_HUMAN	-2.74179
285	28551	6633	H300008112	-	-2.96852
286	27246	7244	OPHSV0400005188	DDI2	-2.60034
287	31911	6462	H300019629	STOML1	-2.19396
288	38057	5481	H300021492	AGPAT7	-2.77085
289	32276	6767	H300007223	OR2AJ1	-2.58841
290	32128	7024	OPHSV0400004417	FAM114A1	-2.13283

291	38029	6028	H300015331	CALD1	-2.61664
292	37373	7032	OPHSV0400004347	-	-3.33847
293	32063	8817	OPHSV0400004655	CYP2B	-2.27703
294	31073	9327	OPHSV0400004538	Q6ZV82_HUMAN	-2.0711
295	33929	8710	H300007217	SPAST	-2.05461
296	38610	8004	OPHSV0400007287	-	-2.35235
297	40571	7649	H200002589	FBXO3	-2.55422
298	39259	8154	H300014161	FCHO2	-2.38137
299	34539	9505	H300007212	SLC27A1	-2.33921
300	30900	11006	H200016568	CLDN14	-2.04201
301	37157	9523	OPHSV0400005288	CN057_HUMAN	-2.70653
302	32505	11138	H200004241	ARCN1	-2.11681
303	36910	10169	OPHSV0400005394	RBM34	-2.85837
304	42148	9623	H300014737	PTHR2	-2.51142
305	42617	9611	OPHSV0400004333	-	-2.53433
306	42312	9815	H300007215	SLC27A1	-2.47701
307	46034	9177	H300014197	FXYD2	-2.79191
308	44147	10904	OPHSV0400004339	Q9P147_HUMAN	-2.53206
309	45054	11155	H300002006	-	-2.27834
310	49306	10252	H200006240	PNRC1	-2.61608
311	55833	9572	H300005205	Q8NI68_HUMAN	-3.00992
312	52138	11230	H300007216	SPAST	-3.24122
313	44748	14361	OPHSV0400005571	Q6ZMM1_HUMAN	-3.08126
314	39839	16470	H200008207	AOF2	-2.54865
315	56283	12738	OPHSV0400005878	TRPV1	-3.80437
316	43070	19758	H300019025	OBP2A	-2.1005
317	44025	20234	H200018878	PCM1	-2.04122
318	48503	18658	H300022214	WHSC1L1	-2.45509
319	48275	20967	H200020201	PNMA5	-2.23381
320	53890	28231	H300005116	NP_940973.1	-2.59869
321	65263	29327	H200004282	BLOC1S2	-2.62972
322	61831	31573	OPHSV0400005260	-	-2.13348
